# Inhibition of glutamine metabolism accelerates resolution of acute lung injury

**DOI:** 10.14814/phy2.14019

**Published:** 2019-02-28

**Authors:** Christine L. Vigeland, Henry S. Beggs, Samuel L. Collins, Yee Chan‐Li, Jonathan D. Powell, Claire M. Doerschuk, Maureen R. Horton

**Affiliations:** ^1^ Department of Medicine University of North Carolina Chapel Hill North Carolina; ^2^ Marsico Lung Institute University of North Carolina Chapel Hill North Carolina; ^3^ Department of Medicine Johns Hopkins University School of Medicine Baltimore Maryland; ^4^ Sidney Kimmel Comprehensive Cancer Center Johns Hopkins University School of Medicine Baltimore Maryland; ^5^ Center for Airways Disease University of North Carolina Chapel Hill North Carolina

**Keywords:** Acute lung injury, amphiregulin, glutamine, inflammation, resolution of injury

## Abstract

Despite recent advances, acute respiratory distress syndrome (ARDS) remains a severe and often fatal disease for which there is no therapy able to reduce the underlying excessive lung inflammation or enhance resolution of injury. Metabolic programming plays a critical role in regulating inflammatory responses. Due to their high metabolic needs, neutrophils, macrophages, and lymphocytes rely upon glutamine metabolism to support activation and function. Additionally, during times of physiologic stress, nearly all cells, including fibroblasts and epithelial cells, require glutamine metabolism. We hypothesized that inhibiting glutamine metabolism reduces lung inflammation and promotes resolution of acute lung injury. Lung injury was induced by instilling lipopolysaccharide (LPS) intratracheally. To inhibit glutamine metabolism, we administered a glutamine analogue, 6‐diazo‐5‐oxo‐L‐norleucine (DON) that binds to glutamine‐utilizing enzymes and transporters, after injury was well established. Treatment with DON led to less lung injury, fewer lung neutrophils, lung inflammatory and interstitial macrophages, and lower levels of proinflammatory cytokines and chemokines at 5 and/or 7 days after injury. Additionally, DON led to earlier expression of the growth factor amphiregulin and more rapid recovery of LPS‐induced weight loss. Thus, DON reduced lung inflammation and promoted resolution of injury. These data contribute to our understanding of how glutamine metabolism regulates lung inflammation and repair, and identifies a novel target for future therapies for ARDS and other inflammatory lung diseases.

## Introduction

Acute respiratory distress syndrome (ARDS) is a severe, often fatal syndrome characterized by excessive lung inflammation and damage to the alveolar‐capillary barrier (Ware and Matthay 2000; Piantadosi and Schwartz 2004). Current treatment for ARDS focuses on supportive care, and there are no treatments able to target the underlying inflammation and alveolar damage (Zeiher et al. 2004; Steinberg et al. 2006; Liu et al. 2008; National Heart et al. 2014). Thus, there is a critical need for understanding mechanisms underlying resolution of lung injury and for novel approaches to modulate the processes that regulate resolution.

Metabolic programming is one of the important mechanisms by which immune responses are regulated. During times of physiologic stress, glutamine utilization increases considerably, and glutamine becomes an essential nutrient (Wilmore and Shabert 1998). In critical illness, a decrease in serum glutamine level is associated with increased mortality (Oudemans‐van Straaten et al. 2001). Thus, glutamine supplementation has been investigated as a treatment for critical illnesses such as ARDS and sepsis. However, results have been heterogeneous. Some animal studies looking at glutamine supplementation in models of acute lung injury (ALI) induced by abdominal sepsis, lipopolysaccharide (LPS), or acid aspiration found that glutamine given ***prior*** to the development of injury or within the first hour of injury attenuated the development of lung injury over the first 24 h (Singleton et al. 2005; Zhang et al. 2009; Lai et al. 2014). In contrast, another study found that glutamine supplementation prior to LPS‐induced ALI led to increased lung injury at both 24 h and 2 weeks (Hou et al. 2009). When critically ill patients were treated with glutamine supplementation, the results have been mixed (Andrews et al. 2011; Wernerman et al. 2011; Heyland et al. 2013). Although the primary outcome is not identical in these studies, one study suggested glutamine did not affect the measured outcomes, one suggested that ICU mortality was less, and the third showed that mortality was actually increased following glutamine supplementation.

One explanation for these seemingly contradictory results is that the role of glutamine may vary depending on the phase of injury. In vitro studies show that glutamine potentiates the activity of inflammatory cells, promoting activation and function of neutrophils and macrophages and proliferation and differentiation of lymphocytes (Wallace and Keast 1992; Wells et al. 1999; Furukawa et al. 2000; Pithon‐Curi et al. 2003; Carr et al. 2010; Nakaya et al. 2014). Thus, in patients with ongoing, excessive inflammation, glutamine may in fact be fueling that inflammatory response and increasing disease severity. Studies in models of other inflammatory diseases have found that inhibiting glutamine metabolism can reduce inflammation, promote immunological tolerance, and improve survival (Gordon et al. 2015; Lee et al. 2015; Potter et al. 2015).

We hypothesized that in ALI, glutamine metabolism promotes excessive inflammation and that inhibition of glutamine metabolism reduces inflammation and promotes resolution of injury. To inhibit glutamine metabolism, we utilized a glutamine analogue, 6‐diazo‐5‐oxo‐L‐norleucine (DON), which binds to and inhibits glutamine‐utilizing enzymes and transporters, blocking glutamine metabolism and uptake into cells (Pinkus 1977). To study resolution of injury, we began administering DON 2 days ***after*** induction of ALI. We found that inhibition of glutamine metabolism with DON accelerated resolution of ALI, reducing lung inflammation and promoting expression of the growth factor amphiregulin (AREG). Our data reveal the critical role of glutamine in promoting inflammation and injury in ALI and identify a novel therapeutic target to accelerate resolution of injury.

## Materials and Methods

### Mice

C57BL/6 mice were purchased from The Jackson Laboratory (Bar Harbor, ME) and housed at the University of North Carolina animal facility. Mice aged 8 to 10 weeks old were utilized for experiments, and each experiment was age and sex matched. Weight was monitored daily, and mice that demonstrated ≥ 25% weight loss for two consecutive days were euthanized. All animal protocols were approved by the University of North Carolina at Chapel Hill School of Medicine Institutional Animal Care and Use Committee.

### Initiation of lung injury

To determine the role of glutamine metabolism in ALI, we utilized an established model of lung injury and repair. Mice were anesthetized with tribromoethanol prior to orotracheal intubation with a 20‐gauge catheter as previously described (Dial et al. 2017). *Escherichia* coli LPS O55:B5 (7.5 mg/kg) (Sigma‐Aldrich, St. Louis, MO) was then instilled intratracheally (IT) via the 20‐gauge catheter. Control mice received an equal volume of vehicle. The mice were immediately extubated and allowed to awaken. DON (1.6 mg/kg, Sigma‐Aldrich, St. Louis, MO) was administered intraperitoneally (IP) on days 2, 4, and 6 following LPS administration (Lee et al. 2015). Control mice received an equal volume of vehicle. At 5 and 7 days after injury, mice were euthanized by isoflurane overdose and exsanguination from the inferior vena cava.

### BAL collection and protein measurement

Bronchoalveolar lavage (BAL) fluid was collected by intubating the trachea with a 20‐gauge catheter and performing two lavages, each with 750 *μ*L of PBS. BAL fluid was centrifuged at 500*g* for 5 min. The supernatant was separated for measurement of protein by *DC*™ protein assay (Bio‐Rad, Hercules, CA) as per the manufacturer's protocol.

### Flow cytometry

Lung tissue was processed to form a single cell suspension and immunostained to identify cell types as previously described (Dial et al. 2017). Antibodies were obtained from Biolegend (San Diego, CA): CD45 FITC (clone 30‐F11), SiglecF PE (clone E50‐2440), Annexin V PerCP‐Cy5.5 (catalog 640936), CD64 PE‐Cy7 (clone X54‐5/7.1), Ly6G APC (clone 1A8), CD3 FITC (clone 145‐2C11), CD45 Alexa Fluor 700 (clone 30‐F11), CD4 PB (clone GK1.5), Foxp3 Alexa Fluor 647 (clone 150D), Zombie Aqua™ (catalog 423102); from BD Horizon (Waltham, MA): Ly6C BV421 (clone AL‐21); and from BD Pharmingen (Waltham, MA): CD16/CD32 Mouse BD Fc block™ (clone 2.462). eBioscience (Waltham, MA) fixation/permeabilization reagents were used. The cells were examined using flow cytometry (Cytoflex, Beckman Coulter, Brea CA) and analyzed using CytExpert (Beckman Coulter) software.

### Quantitative RT‐PCR

Cell pellets of homogenized lung tissue were snap‐frozen at −80°C, and RNA was extracted using Qiagen miRNeasy Mini Kit (Hilden, Germany) according to the manufacturer's protocol. cDNA was produced using the Bio Rad iScript cDNA synthesis kit per the manufacturer's instructions. qRT‐PCR was performed, using PrimeTime Gene Expression Master Mix (Integrated DNA Technologies, Skokie, IL) on a QuantStudio 6 Flex (Applied Biosystems by Life Technologies, Waltham, MA) and normalized to 18s (Life Technologies). Taqman probes were purchased from Thermo Fisher Scientific (Waltham, MA) for TNF‐*α*, IL‐6, IL‐1*β*, AREG, KGF, CXCL1, and CCL2.

### Histology

Lungs were inflated with formalin to 22 cm H_2_O atmospheric pressure. Tissue blocks were embedded in paraffin, sectioned at 4–5 *μ*m, and stained with H&E stain by the UNC Animal Histopathology & Lab Medicine Core.

### Statistics

Statistical analysis was performed using GraphPad Prism 7.03 software (La Jolla, CA). Mortality differences were analyzed by the log‐rank test. To compare means between two groups, Student's t‐tests with Holm‐Sidak correction were performed. To compare more than two groups, two‐way ANOVA was used, followed by Tukey's, or Dunnett's correction for multiple comparisons. Statistical difference was accepted at *P *<* *0.05.

## Results

### Inhibition of glutamine metabolism with DON accelerates resolution of established ALI

**Figure 1:**
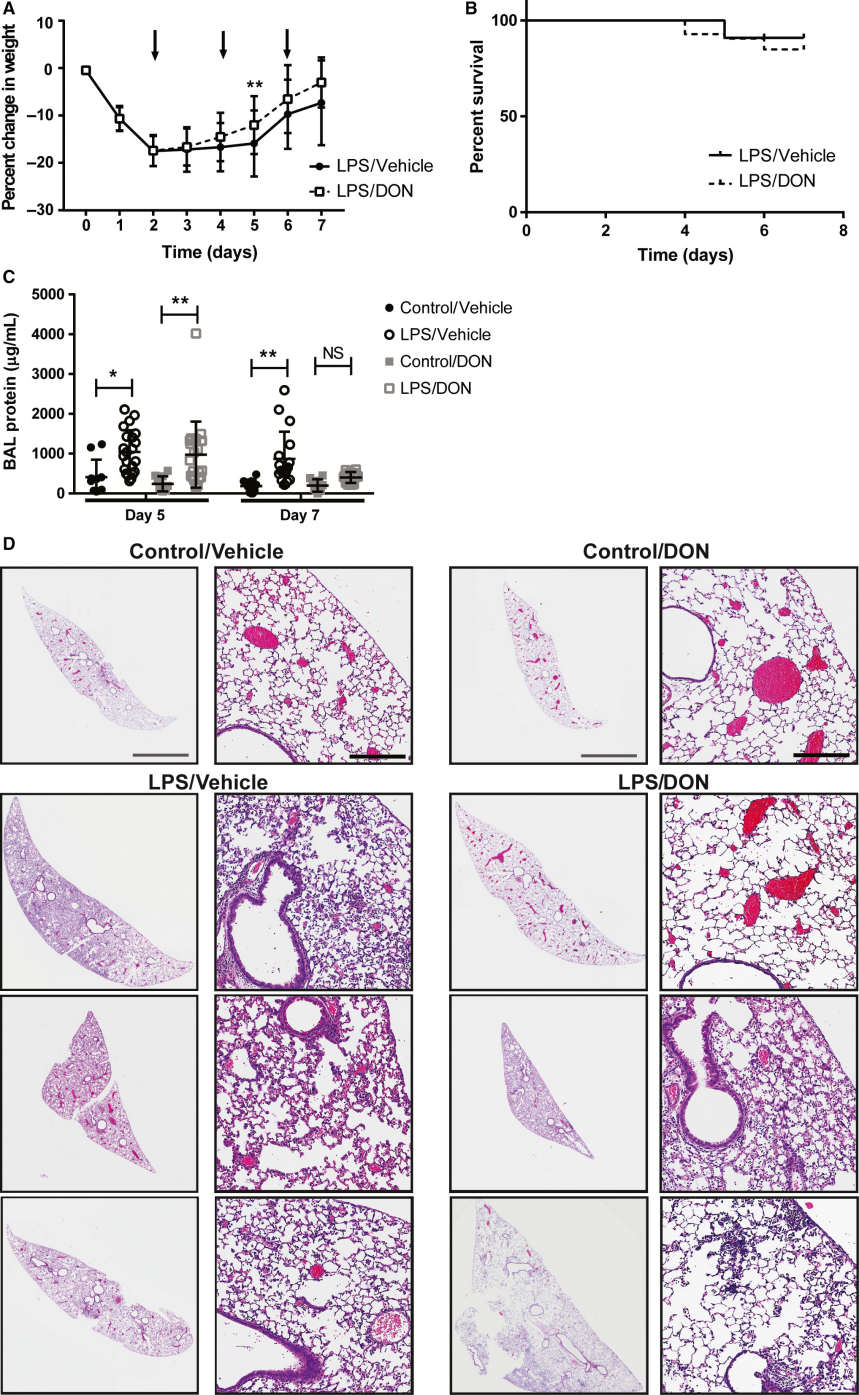
Inhibition of glutamine metabolism with DON accelerates resolution of established ALI. C57BL/6 mice (*N* = 20 per control group, 42–44 per LPS group) received LPS (7.5 mg/kg IT) or vehicle control on day 0 to induce ALI, followed by DON (1.6 mg/kg IP) or vehicle on days 2, 4, and 6 (indicated by arrows). (A) Change in weight from baseline following LPS. Significance was determined by multiple t‐tests with Holmes–Sidak correction. **P < 0.01 compared to mice given LPS followed by vehicle. (B) Survival curve following LPS‐injury. *P *=* *0.64 by Log‐rank test. (C) BAL protein on days 5 and 7 following LPS. *N* = 10 per control group and 15‐22 per LPS group. Error bars represent one standard deviation. Significance was determined by two‐way ANOVA followed by Tukey's multiple comparisons test. *P < 0.05, **P < 0.01. (D) Representative samples of H&E‐stained lung histology from uninjured mice and three DON‐ and three vehicle‐treated injured mice at day 5 (*n* = 5 per treatment). Gray scale bar indicates 2 mm; black‐scale bar indicates 200 *μ*m.

C57BL/6 mice received LPS or vehicle control on day 0 to induce ALI. Mice then received either DON or vehicle on days 2, 4, and 6. Following LPS administration, both DON‐ and vehicle‐treated mice lost a significant amount of weight (Fig. 1A). For vehicle‐treated mice, weight loss persisted through days 2–5, and mice began to gain weight on days 6–7. However, DON‐treated mice demonstrated accelerated recovery of body weight, with significantly greater weight on day 5 and a trend toward greater weight on day 7 (*P *=* *0.06). There was no difference in survival between the DON‐treated and untreated groups (*P *=* *0.64) (Fig. 1B). Weight data for mice that did not survive were included until the time of death; however, other data were not included in the analysis.

Following LPS injury, there was increased protein in BAL fluid on days 5 and 7; however, in the DON‐treated mice, BAL protein was only elevated on day 5, and there was no significant elevation in BAL protein on day 7 (Fig. 1C). Lung histology was assessed on day 5 following LPS injury, and DON‐treated mice demonstrated less lung inflammation by H&E staining (Fig. 1D).

### Inhibition of glutamine metabolism with DON in established ALI reduces lung inflammatory cells

**Figure 2:**
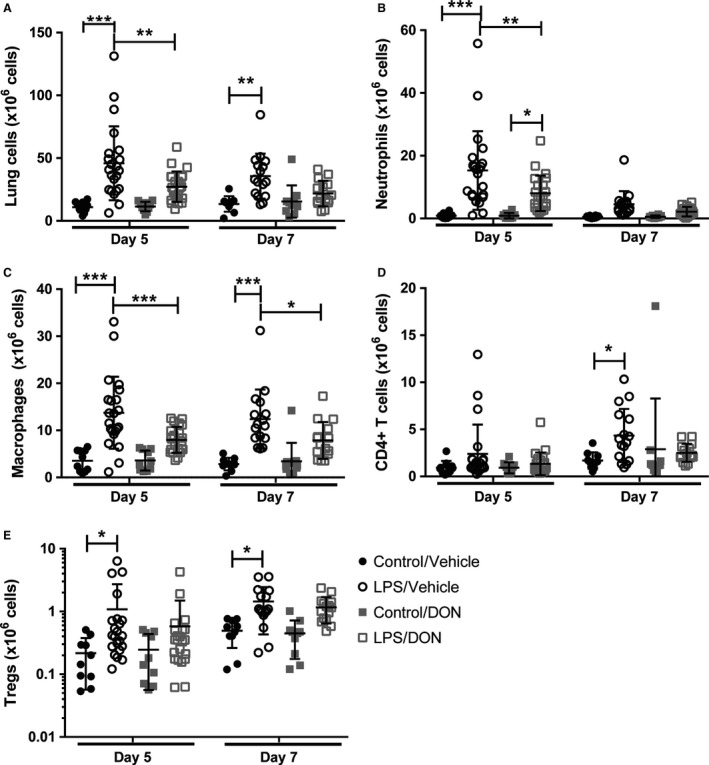
Inhibition of glutamine metabolism with DON in established ALI reduces lung inflammatory cells. Lungs were analyzed on days 5 and 7 by flow cytometry to determine the number of (A) lung cells, (B) neutrophils, (C) macrophages, (D) CD4+ T cells, and (E) Tregs. *N* = 10 per control group and 15–22 per LPS group. Error bars represent one standard deviation. Significance was determined by two‐way ANOVA followed by Tukey's multiple comparisons test (A–C) or Dunnett's multiple comparisons test (D–E). *P < 0.05, **P < 0.01, ***P < 0.001.

We characterized the inflammatory infiltrate within the lungs by flow cytometric analysis of immunostained lung digests. On days 5 and 7 following LPS administration, there was an increase in the number of cells within the lungs, primarily neutrophils (CD45 + Ly6G+) and macrophages (CD45+Ly6G‐CD64+) on day 5 and macrophages and CD4+ T cells (CD45+CD3+CD4+) on day 7 (Fig. 2A–D). Treatment with DON beginning at 48 hours after initiation of ALI, when inflammation and injury are well established, resulted in fewer lung neutrophils on day 5 and fewer macrophages on days 5 and 7. There was no statistical difference in the number of CD4 + T cells between mice receiving DON or vehicle after LPS injury. In contrast, the number of lung regulatory T cells (CD45+CD4+Foxp3+) increased on days 5 and 7 following LPS; however, this increase was not affected by DON treatment (Fig. 2E).

**Figure 3:**
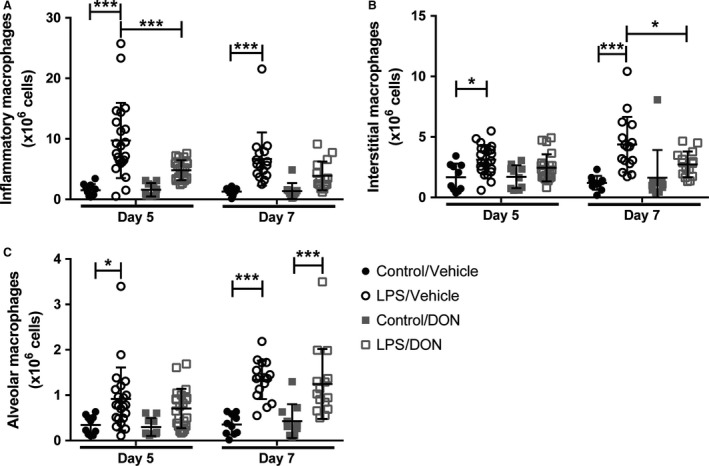
Inhibition of glutamine metabolism with DON in established ALI alters lung macrophage phenotypes. Lungs were analyzed on day 5 and 7 by flow cytometry to quantify (A) inflammatory macrophages, (B) interstitial macrophages, (C) alveolar macrophages. *N* = 10 per control group and 15–22 per LPS group. Error bars represent one standard deviation. Significance was determined by two‐way ANOVA followed by Tukey's multiple comparisons test. *P < 0.05, **P < 0.01, ***P < 0.001.

We then further characterized lung macrophages by flow cytometry, identifying alveolar macrophages (CD45 + Ly6G‐CD64 + SiglecF+), inflammatory macrophages (CD45+Ly6G‐CD64+Ly6C+), and interstitial macrophages (CD45+Ly6G‐CD64+Ly6C‐SiglecF‐). Following LPS injury, there was an increase in the number of all three macrophage subpopulations on days 5 and 7 (Fig. 3). DON treatment resulted in fewer inflammatory macrophages on day 5 and fewer interstitial macrophages on day 7 (Fig. 3A–B). DON did not affect the number of alveolar macrophages following LPS injury (Fig. 3C).

### DON alters expression of cytokines, chemokines, and growth factors within the lungs

**Figure 4:**
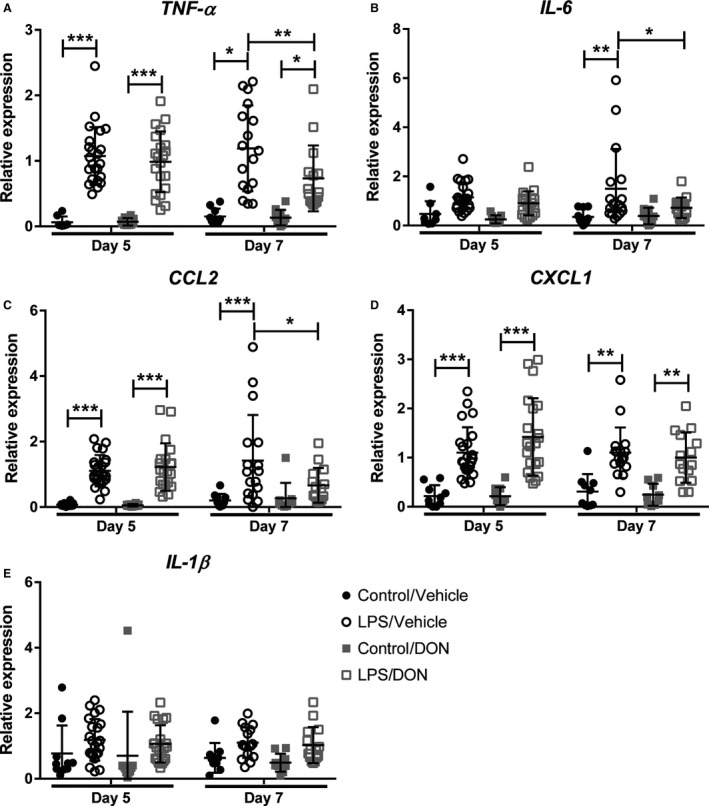
DON alters expression of cytokines, chemokines, and growth factors when given to established ALI. mRNA expression of cytokines and chemokines within lung tissue by qRT‐PCR on days 5 and 7 following LPS administration and DON treatment. (A) TNF‐*α*, (B) IL‐6, (C) CCL2, (D) CXCL1, (E) IL‐1*β*. *N* = 10 per control group and 15–22 per LPS group. Error bars represent one standard deviation. Statistical significance was determined with two‐way ANOVA followed by Tukey's multiple comparisons test. *P < 0.05, **P < 0.01, ***P < 0.001.

In addition to characterizing the inflammatory infiltrate by flow cytometry, we measured mRNA expression of proinflammatory cytokines and chemokines within lung tissue by qRT‐PCR to characterize further the inflammatory response following LPS and to determine the impact of DON on these mediators. LPS increased expression of genes coding for TNF‐*α*, IL‐6, CXCL1, and CCL2 at days 5 and 7 but did not impact on the expression of IL‐1*β* mRNA (Fig. 4A–E). DON resulted in lower levels of expression of TNF‐*α*, IL‐6, and CCL2 on day 7. DON had no impact on the levels of IL‐1*β* or CXCL1.

**Figure 5:**
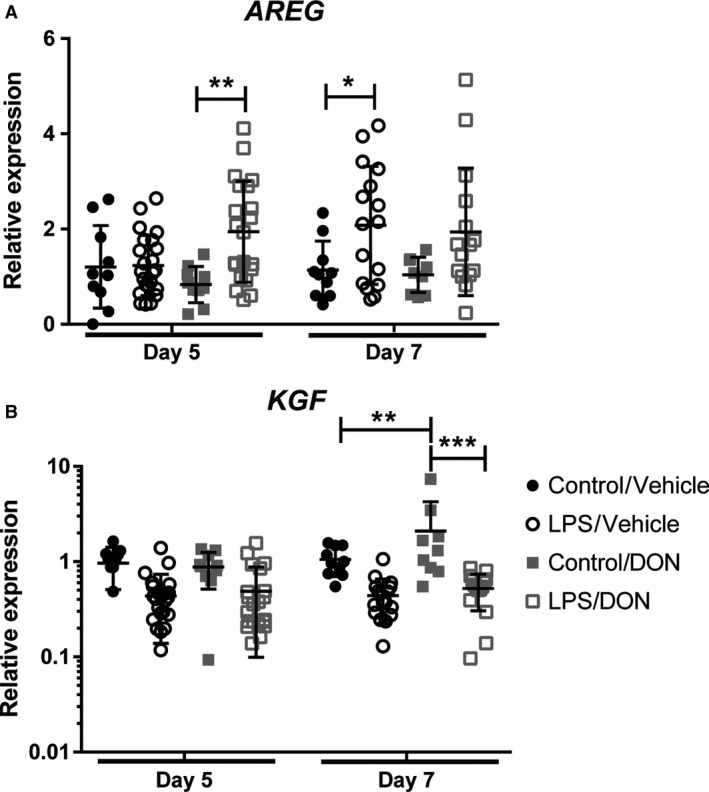
DON promotes expression of AREG but not KGF when given to established ALI. mRNA expression of (A) AREG and (B) KGF within lung tissue by qRT‐PCR on days 5 and 7 following LPS‐induced ALI and DON treatment. *N* = 10 per control group and 15‐22 per LPS group. Error bars represent one standard deviation. Statistical significance was determined with two‐way ANOVA followed by Dunnett's multiple comparisons test (A) or Tukey's multiple comparisons test (B). *P < 0.05, **P < 0.01, ***P < 0.001.

To determine the mechanism through which DON may be acting to aid resolution of lung injury, we measured mRNA expression of two growth factors that have been shown to be important in resolution of ALI: AREG and keratinocyte growth factor (KGF) (Shyamsundar et al. 2014; Xu et al. 2016; Ogata‐Suetsugu et al. 2017). After LPS administration, expression of AREG within the lungs increased on day 7 (Fig. 5A). With DON treatment, this increase in AREG expression occurred earlier, on day 5. In contrast, KGF expression did not change following LPS injury; however, it was increased in uninjured mice receiving DON from days 2–7 (Fig. 5B).

## Discussion

Metabolic programming is an important regulator of inflammatory responses. Our study reveals the critical role of glutamine in regulating lung inflammation and repair in ALI. Prior in vitro work has shown that glutamine metabolism promotes activation and function of neutrophils and macrophages and proliferation and differentiation of lymphocytes (Wallace and Keast 1992; Wells et al. 1999; Furukawa et al. 2000; Pithon‐Curi et al. 2003; Carr et al. 2010; Nakaya et al. 2014). In fact, glutamine is utilized by nearly all cell types, and their relative need for glutamine and its metabolites may vary. We hypothesized that in LPS‐induced ALI, glutamine promotes inflammation and that inhibiting glutamine metabolism attenuates the inflammatory response and accelerates lung injury resolution.

Our study found that inhibition of glutamine metabolism with DON accelerated recovery from LPS‐induced ALI, as documented by accelerated weight recovery, less protein in BAL fluid, and less lung inflammation. Further, DON altered the inflammatory response, reducing the number of neutrophils, inflammatory macrophages, and interstitial macrophages; reducing expression of the proinflammatory cytokines and chemokines TNF‐*α*, IL‐6, and CCL2; and promoting expression of the prorepair growth factor AREG. These data support our hypothesis and reveal the important role that glutamine plays in regulating inflammation.

Our data suggest that inhibition of glutamine metabolism with DON accelerates resolution of established LPS‐induced ALI due to both dampening inflammation and enhanced resolution of injury. Prior in vitro studies have shown that glutamine regulates neutrophil and macrophage activity. Glutamine supplementation delays neutrophil apoptosis, enhances production of reactive oxygen species, and is necessary for phagocytosis (Furukawa et al. 2000; Pithon‐Curi et al. 2003). Within macrophages, glutamine promotes production of inflammatory cytokines such as TNF‐*α* and IL‐6 (Wallace and Keast 1992; Wells et al. 1999). In contrast, Tregs do not require glutamine metabolism (Nakaya et al. 2014), and we found no difference in the number of Tregs within the lungs with DON treatment. Furthermore, glutamine does not impact on alveolar macrophages, an anti‐inflammatory macrophage subpopulation that decreases in number during the acute inflammatory response but then recovers. Thus, DON appears to alter the inflammatory milieu of the lung, affecting proinflammatory macrophages and neutrophils while not impacting Tregs, which mediate resolution of ALI (D'Alessio et al. 2009). Whether glutamine inhibition acts solely to dampen the function and clearance of inflammatory cells or whether this inhibition also acts to suppress recruitment of any additional neutrophils and inflammatory macrophages from the circulation at days 3–5 remains to be determined.

One of the other effects of DON found in our study was accelerated production of AREG. AREG is a growth factor in the epidermal growth factor family that promotes tissue repair following injury (Zaiss et al. 2013). In models of lung injury, it is produced by macrophages, regulatory T cells, and type 2 innate lymphoid cells (Monticelli et al. 2011; Arpaia et al. 2015; Xu et al. 2016). Prior studies have found that AREG promotes epithelial repair in LPS‐induced ALI and influenza infection, and in vitro studies have shown that it enhances regulatory T cell activity (Monticelli et al. 2011; Zaiss et al. 2013; Arpaia et al. 2015; Xu et al. 2016; Ogata‐Suetsugu et al. 2017). In fact, administration of an antibody to neutralize AREG worsens LPS‐induced ALI, whereas administration of exogenous AREG ameliorates lung injury (Xu et al. 2016; Ogata‐Suetsugu et al. 2017). Thus, by increasing expression of AREG more rapidly after injury, we postulate that DON enhances resolution of ALI.

In conclusion, metabolic programming is an important regulator of inflammatory responses. Our study reveals the important role of glutamine metabolism in regulating lung inflammation and resolution. To our knowledge, this is the first study to demonstrate that inhibiting glutamine metabolism after inflammation and injury are established leads to accelerated resolution of ALI by reducing lung inflammation and promoting production of the growth factor AREG. These data contribute to our understanding of how glutamine metabolism regulates lung inflammation and repair, and identifies a novel target for future therapies for ARDS and other inflammatory lung diseases.

## Conflict of Interest

J.D.P. is a scientific founder with equity and a paid consultant for Dracen Pharmaceuticals.
